# Independent Lung Ventilation-Experimental Studies on a 3D Printed Respiratory Tract Model

**DOI:** 10.3390/ma14185189

**Published:** 2021-09-09

**Authors:** Katarzyna Kramek-Romanowska, Anna M. Stecka, Krzysztof Zieliński, Agata Dorosz, Piotr Okrzeja, Marcin Michnikowski, Marcin Odziomek

**Affiliations:** 1 Nalecz Institute of Biocybernetics and Biomedical Engineering, Polish Academy of Sciences, Ks Trojdena 4, 02-109 Warsaw, Poland; astecka@ibib.waw.pl (A.M.S.); kzielinski@ibib.waw.pl (K.Z.); pokrzeja@ibib.waw.pl (P.O.); mmichnikowski@ibib.waw.pl (M.M.); 2 Faculty of Chemical and Process Engineering, Warsaw University of Technology, Waryńskiego 1, 00-645 Warsaw, Poland; agata.dorosz.dokt@pw.edu.pl (A.D.); marcin.odziomek@pw.edu.pl (M.O.)

**Keywords:** 3D-printed anatomical model, independent lung ventilation, mechanical ventilation, endotracheal tube, lung compliance, pulmonary flow

## Abstract

Independent lung ventilation (ILV) is a life-saving procedure in unilateral pulmonary pathologies. ILV is underused in clinical practice, mostly due to the technically demanding placement of a double lumen endotracheal tube (ETT). Moreover, the determination of ventilation parameters for each lung in vivo is limited. In recent years, the development of 3D printing techniques enabled the production of highly accurate physical models of anatomical structures used for in vitro research, considering the high risk of in vivo studies. The purpose of this study was to assess the influence of double-lumen ETT on the gas transport and mixing in the anatomically accurate 3D-printed model of the bronchial tree, with lung lobes of different compliances, using various ventilation modes. The bronchial tree was obtained from Respiratory Drug Delivery (RDD Online, Richmond, VA, USA), processed and printed by a dual extruder FFF 3D printer. The test system was also composed of left side double-lumen endotracheal tube, Siemens Test Lung 190 and anesthetic breathing bag (as lobes). Pressure and flow measurements were taken at the outlets of the secondary bronchus. The measured resistance increased six times in the presence of double-lumen ETT. Differences between the flow distribution to the less and more compliant lobe were more significant for the airways with double-lumen ETT. The ability to predict the actual flow distribution in model airways is necessary to conduct effective ILV in clinical conditions.

## 1. Introduction

Independent lung ventilation (ILV) is a life-saving procedure, especially in unilateral pulmonary pathologies that occur, e.g., in lung cancer disease or after severe chest injuries when both lungs have significantly different biomechanical properties [1]. In such cases, each lung needs to be ventilated independently in order to avoid damage to the more affected lung and to speed up its recovery, while allowing the other lung to ventilate properly. ILV requires the use of a left or right sided double-lumen endotracheal tube (ETT) and in most cases, it is carried out using two ventilators not always synchronized [2]. Our current project (LIDER/19/0107/L-8/16/NCBR/2017) is focused on the development of a device for synchronous ILV, design for work with only one ventilator. In addition to its intended purpose, in emergency situations, under ventilators shortage it could be used to support two patients with a single ventilator. This non-standard use appears to be particularly important during the ongoing COVID-19 pandemic [3].

In vivo determination of the realistic parameters of ventilation for each lung is limited. For this reason, an accurate model of the upper part of respiratory system is needed in order to test chosen prerequisites correctly. Typically, in vitro studies concerning mechanical ventilation are conducted in systems not taking into account the geometry of anatomical structures [4]. This constitutes a considerable drawback as the physiological relevance of obtained results may be significantly impaired.

What is more, according to both numerical [5] and in vitro [6,7] studies during mechanical ventilation secondary flow structures were observed in realistic bifurcated geometry as compared to idealized one. In addition, it was observed that either compliance or resistance asymmetry between adjacent lung branches causes secondary flow transport by pendelluft mechanism [8], i.e., asynchronous gas exchange between respiratory units. The pendelluft mechanism is time constant (τ) dependent (τ = Resistance x Compliance); the asynchrony of the time constant due to variation of the respiratory units’ resistance or compliance results in gas exchange between the units [9]. Compliance is the measure of the ease of inflation, the ease of distensibility (Compliance ∆V/∆P), while the resistance can be described as the measure of the flow hindrance imposed by the airways and the viscoelastic forces within the lungs and chest wall that counteract tissue deformation during breathing [10]. Both parameters provide basic and essential information on flow physics that can be used to formulate the ventilation technique also in clinical conditions. 

Besides, various studies [11,12,13,14] dedicated to the prediction of flow phenomena have also concluded that invasive devices like endotracheal tubes during ventilation therapy cause a high-speed jet, which is released at the carina–trachea environment and induces shear stress at the airways walls. The wall shear stress (WSS) is seen to cause airways inflammation and epithelial erosion [12,13]. Considering this, it is clear that models close to real geometry are highly required in the studies concerning air distribution during conventional mechanical ventilation in general and ILV in particular. As a consequence, it is possible both to reduce animal trials and to accelerate the implementation of developed guidelines to clinical practice.

Until recently, obtaining a detailed model of the respiratory tract has posed a real challenge among others due to the complex geometry of this system, intersubject variability observed in computed tomography (CT) scans that hindered model unification, lack of relevant manufacture technique to reproduce the geometry with high precision. However, in the past years, the rapid development of CAD software and 3D printing techniques enabled the manufacture of highly accurate physical models of anatomical structures. This application is particularly important for medical purposes such as pre-treatment training which allows planning and practice complex procedures before performing them in clinical practice [15,16,17] and the production of patient-specific biocompatible implants [18,19]. Another significant application is the possibility of running in vitro tests to determine the influence of external factors on selected anatomical structures [20].

An equally important tool to study the role of mechanical ventilation on pulmonary air flow is computational fluid dynamics (CFD). Numerical simulations outline fluid behavior and flow structures that cannot be detected either by experimental or in vivo tests. Due to advancements in medical imaging and current computational resources available, it is possible to simulate in a reasonable time the fluid dynamics in models of the respiratory tract based on CT scans of patients [14]. Combining experiments with CFD offers findings which may be particularly important for optimizing ILV management strategies.

According to our knowledge, there is a lack of studies with models reflecting the real geometry of the bronchial tree and double-lumen endotracheal tube under conditions typical for ILV. Therefore, the purpose of this study was to assess the influence of double-lumen ETT on the distribution of pressure and flow in an anatomically accurate 3D-printed model of the bronchial tree, with lung lobes of different compliances, using various ventilation modes. In addition, the CFD technique was employed to perform preliminary simulations in the same model bronchial tree and double-lumen ETT. Some important observations were made concerning the impact of ETT and lung compliance on the gas transport and mixing during ILV. The ability to predict the actual flow distribution in model airways is necessary to conduct ILV effectively in clinical conditions.

## 2. Materials and Methods

### 2.1. Model Preparation and 3D Printing

The computer STereoLithography model of trachea and three consecutive bronchial bifurcations in medium size (reconstructed from computed tomography images) was obtained from Respiratory Drug Delivery (RDD Online, Richmond, VA, USA). The model was imported from the RDD website (https://www.rddonline.com/rdd/rdd.php?sid=105, last access 30 July 2021) from the Aerosol Testing Equipment section. Detailed descriptions of the model have been given elsewhere [21,22]. RDD provides several airway models that represent characteristic adult geometries of the upper tracheobronchial airways based on literature values and medical images. These models can be used to test aerosol deposition, flow field characteristics, or for the construction of computational fluid dynamics (CFD) geometries. The medium tracheobronchial (TB) model, utilized throughout the study, constitutes an original TB geometry based on the measurements applied to physiologically realistic, asymmetrical bifurcation units and scaled to a functional residual capacity (FRC) of 3.5 L. The bifurcation units were rotated in out of plane to approximate the gravity angles. The second bifurcation (B2) of the left lung feeds the left upper and left lower lobes. In the right lung, B2 feeds the right upper lobe directly, whereas the lower third bifurcation feeds the right middle and right lower lobes. 

**Figure 1:**
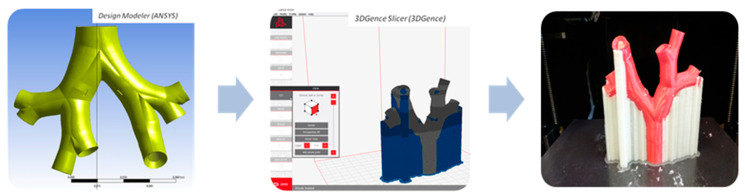
Consecutive steps of model respiratory tract preparation.

The model was imported to Design Modeler provided by ANSYS to adjust the geometry for the 3D printing (see Figure 1).

Due to the complex internal geometry (e.g., narrow ducts), the model was prepared with the aid of 3DGence Slicer and printed by the dual-extruder printer Double P255 (3DGence, Przyszowice, Poland). This complex geometry has been produced with two separate materials for the base model and support. In order to achieve good print properties (among others negligible material contraction, easy support removal and no model damage after support removal), we used: polylactic acid (PLA, 3DGence, Przyszowice, Poland) for the base and water-soluble Butenediol Vinyl Alcohol Co-polymer (BVOH) for the support. In this study, the main aim was to recreate the details of complex geometry, not to imitate material properties of the airway walls. Printing settings are summarized in Table 1.

After dissolution of the support material, we obtained the base model without any post-processing damages to its internal surface. 

### 2.2. Experimental Setup

**Figure 2:**
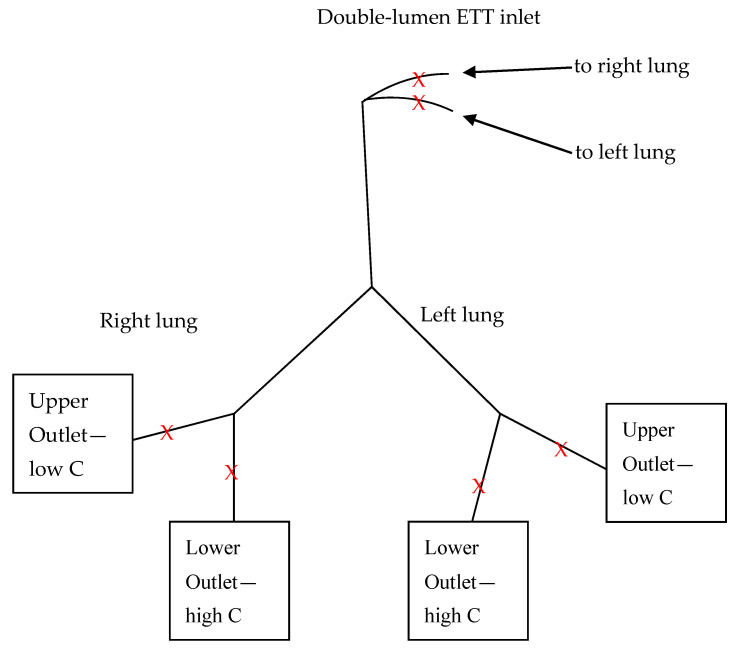
Scheme of the model respiratory system used in the study: C—compliance, ETT—endotracheal tube, Lower Outlet—anesthetic breathing bag, Upper Outlet—Siemens test lung, X—measurement point.

During model post-processing, to reduce the number of experimental variants, a truncated model of the respiratory system was prepared that encompassed trachea and the first two generations. All next generations (third and above) were cut, and in total 4 outlets were obtained, as presented schematically in Figure 2.

**Figure 3:**
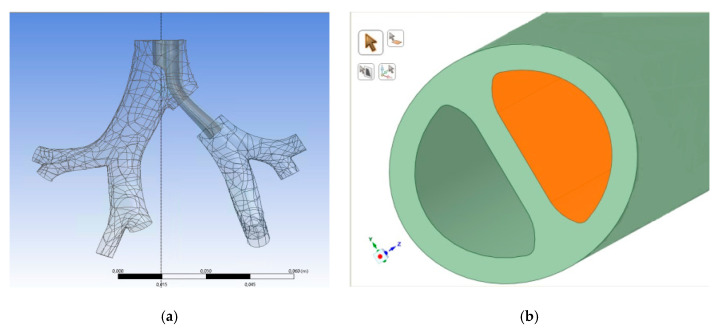
The location of the ETT end in the model of tracheobronchial tree and the cross section of the tube. (**a**) Computer model of ETT end position in the tracheobronchial tree; (**b**) cross section of double-lumen ETT computer model.

Left side double-lumen ETT—endotracheal tube (type Bronchopart, Rusch) size 28, fitting the RDD model of the tracheobronchial tree was donated by Teleflex Inc. (Wayne, PA, USA). The location of the ETT end in the model of the tracheobronchial tree was presented in Figure 3, as well as the cross-section of the tube.

Siemens Test Lung 190 (Siemens AG, Munich, Germany) and anesthetic breathing bag (Wdimed, Poland) were used as model lung lobes with different compliances. Mechanical ventilation with various tidal volume divisions was performed by the Ventilator Nellcor Puritan Bennett 840 (Covidien, Minneapolis, MN, USA) according to variants listed in Table 2.

System static compliance was measured by the ventilator. The physiological value for compliance is 100 to 200 mL/cm H_2_O. Intubation reduces this number to approximately 100 mL/cm H_2_O, while values close to 200 mL/cm H_2_O are an indication of pathology, e.g., emphysema. Changes in system compliance were realized by adjusting elastic properties of the anesthetic breathing bag (model lung lobe with higher compliance). Air mass flow was measured by Mass Flow Meter SFM3000 (Sensirion, Switzerland), while for pressure monitoring the manometer was used with a 50 Hz sampling rate, constructed by IBBE PAS (Institute of Biocybernetics and Biomedical Engineering, Polish Academy of Sciences, Warsaw, Poland). The manometer is an enhanced version of the device (IBBE PAS) dedicated to pleural pressure measurement and has been successfully used in over 60 patients up to the present [23,24]. The main part of the device is the Smiths Medical DPT-8003 pressure transducer, and its measuring range is equal to □+/− 300 cm H_2_O). The electrical part of the manometer consists of the AD7730, Atmega8A and FT230XS chips, which send the electrical signal to the computer equipped with the software that enables calibration, measurement display and collection of the pressure signal.

Pressure (P) and flow (Q) measurements were done for ca. 10 respiratory cycles in 3 measuring points for both sides, i.e., ETT inlet, upper outlet—just before the Siemens test lung, lower outlet—just before the anesthetic bag (as depicted in Figure 2). The pressure-time and flow-time relationships were averaged over time of the respiratory cycle.

### 2.3. Numerical Simulations

ANSYS Kampus 19.1 Licence was applied for the geometry preparation and further numerical operations. The computational domain encompassed the fluid domain inside the final end of the ETT (right air duct), the region of the first bifurcation below upper cuff, over carina and over lower cuff, as well as the fluid domain inside the second generation of the tracheobronchial tree. The additional extension of the domain was created to maintain a fully developed velocity profile at the end of the ETT instead of a uniform velocity distribution. Additional segments were created based on the real dimensions of the ETT, assuring transition from the circular cross-section (7 mm in diameter) to the desired, final moon-shaped one: (1) cylindrical region (diameter: 7 mm, height: 55 mm); (2) region of a variable cross-section (transition from circular profile (diameter: 7 mm) to moon-shaped profile, height: 23 mm); (3) region of a moon-shaped cross-section (height: 200 mm). To avoid any backflow due to an abrupt end of the geometry, the necessary artificial elongations of the bronchi (of a constant cross-section) were also added.

The commercial computational fluid dynamic package ANSYS CFD and ANSYS Fluent solver was used to compute the air flow through the domain using chosen steady-state conditions, representative to the peak inlet flow of the inspiratory pattern (Vt = 375 mL, division 3:1 (normal compliance ca. 100)). Flow conditions were assumed to be isothermal and incompressible. The working fluid for all simulations reported in this study was air at 36.6 °C (ρ = 1.14 kg/m^3^; μ = 1.899 × 10^−5^ kg/m s). To model turbulence, shear stress transport (SST) model was used, as it performs well for boundary layer flows, with low Reynolds correction. The shear-stress transport (SST) k-omega was developed to adequately blend the accuracy and robustness of the k-omega model in the near-wall region while preserving the free-stream independence of the k-epsilon model in the far field. 

As utilizing of STL geometries, such as RDD model, requires the implementation of tetrahedral mesh elements, to study mesh grid sensitivity, three tetrahedral meshes were adopted: coarse (1.9 × 10^6^ cells), medium (2.0 × 10^6^ cells), and fine (2.2 × 10^6^ cells) of good quality. The meshes were assessed based on obtained values of aspect ratio, skewness and orthogonal quality. To obtain mesh-independent results in reasonable amount of time, the optimal, sufficiently dense mesh was medium one with number of 2.0 × 10^6^ cells. Mesh density was locally increased in the following regions: (1) near the bifurcation points (two carinas); (2) in the regions below the upper cuff and above the lower cuff of the ETT—in the peripheral ridges; (3) near the outlet of the ETT.

The walls of the domain were assumed to be smooth, dry, and rigid imposed. A no-slip boundary condition was applied to the walls of the domain—tracheobronchial airway walls and the surface of the cuffs of the ET tube. Based on the peak value of the inlet flow rate, a mass flow inlet condition was assumed at the inlet of the extended ET tube equal to 0.19323 kg/s, based on its circular diameter. To obtain physiologically relevant boundary conditions, the pressure at each outlet (upper—right apical and lower—right main) must be known a priori. Based on own experimental results (not presented here), the pressure specification was then as follows: 6.91 cm H_2_O corresponding to 677.15 Pa at the upper outlet and 7.12 cm H_2_O corresponding to 698.03 Pa at the lower outlet of the domain. For the pressure equation, as well as convection-diffusion equations, a second order upwind scheme was used to interpolate values from cell centers to nodes. Convergence was determined based on mass, momentum and energy residuals below 1 × 10^−4^ at the end of each update.

## 3. Results and Discussion

### 3.1. Impact of Double-Lumen ETT

In the first step, the impact of double-lumen ETT on the airway resistance (measured by the respirator) and flow distribution in the model tracheobronchial tree was investigated. Table 3 provides a comparison of resistance results and pressure and flow ratios obtained for both sides of the tracheobronchial tree with and without ETT for indicated V_T_ divisions (static system compliance was equal to ca. 100 mL/cm H_2_O). Pressure and flow ratios were calculated for averaged maximum values of these parameters reached during inspiration phase.

Analyzing Table 3, it can be seen that in the case of the tree with double-lumen ETT, the measured resistance increased six times as compared to tree without ETT and is 2–3 times higher than it is commonly expected in the case of a single-lumen ETT [14]. Normal adult airway resistance ranges from approximately 0.5 to 2.5 cm H_2_O/L/s. In a healthy adult intubated with an 8.0 mm ETT, airway resistance would be 4 to 6 cm H_2_O/L/s higher than this normal range due to the additional resistance imposed by the tube. Besides, marked differences between the flow distribution to the LO (high compliance lobe) and UO (low compliance lobe) were observed for both lungs, no matter whether the tube was present or not. Nevertheless, the phenomenon turned out to be more significant for the airways with double-lumen ETT than without ETT. The flow was distributed in the following way—the high compliance lobe got more air during the breath cycle than the low compliance one and the factor was in the range 1.68–1.85 for the case without ETT and in the range 1.86–1.96 for the case with ETT. At the same time, no significant changes (coefficient of variation less than 1.0%, detailed data not presented here) in pressure results were observed. Consequently, it was decided to concentrate only on the flow variations in the further analysis presented in this study.

**Figure 4:**
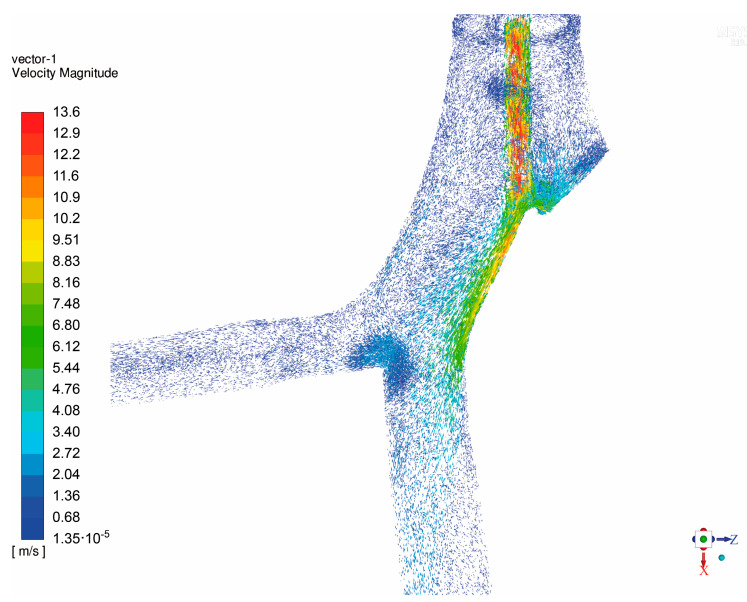
The air velocity profile obtained in the analyzed computational domain during inhalation phase (steady air flow assumed).

The asymmetric form of the tube cross-section, as depicted in Figure 3, may also have played an important role in the observed flow phenomena. At the double-lumen ETT outlet certain flow structures can be expected such as fluid jet, increased turbulence and recirculation zone with high wall shear stress, as was observed for single-lumen tube [11,14]. The nature of the effect may depend on the positioning of the ETT tube, as well. In order to confirm this hypothesis, CFD simulation was performed in the domain described in Section 2.3. In Figure 4, the air velocity profile obtained in the analyzed computational domain during the inhalation phase (steady air flow assumed) is presented.

As one can easily notice, a considerable high-speed jet caused by the ETT was observed at the tube right outlet, as well as the impingement at the carina ridge, which is in line with the previously mentioned study [14]. The increased turbulence and recirculation zone were found to strongly influence the flow distribution in the same way as it was detected during experimental tests—more air is distributed to the lower lobe (outlet) than to the upper one. Noteworthily, the occurrence of high wall shear stress at the impingement sites is considered to be a risk to the wall epithelium layer as it may result in damage and inflammation that leads to ventilator-induced lung injury [25].

### 3.2. Impact of System Compliance

**Figure 5:**
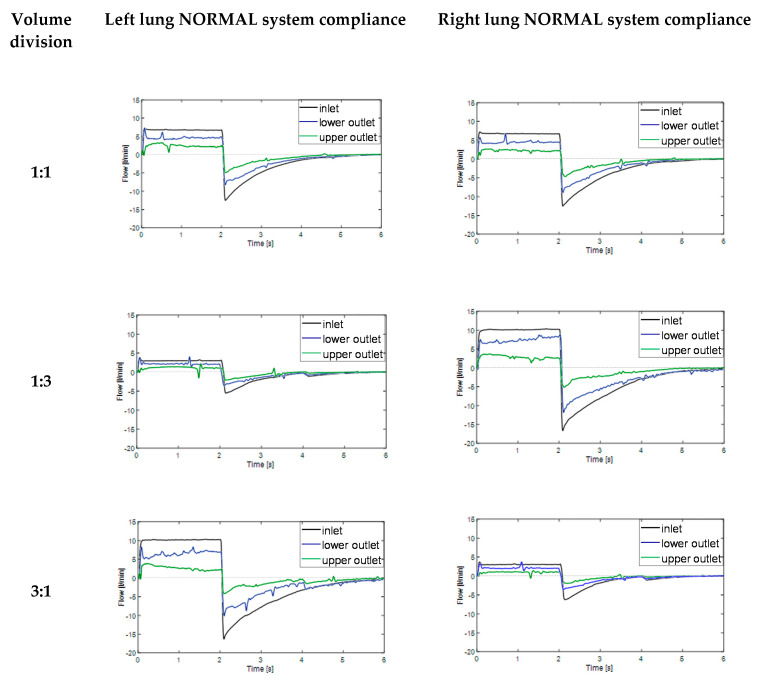
Flow-time relationships during breath cycle for model lungs with normal compliance (ca. 100 mL/cm H_2_O) for various tidal volume divisions (lower outlet—branch with higher compliance; upper outlet—branch with lower compliance). Time 0–2 s represents inspiration phase, time 2–6 s represents expiration phase.

**Figure 6:**
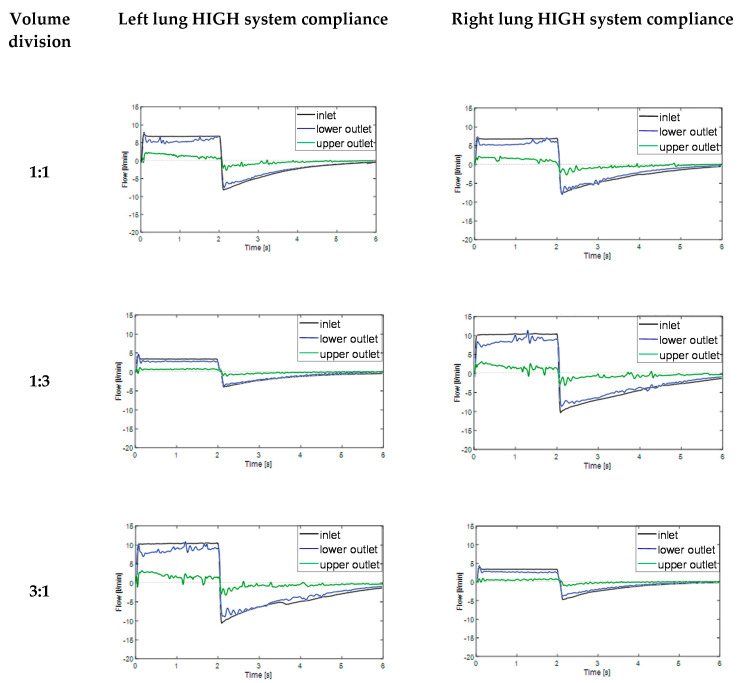
Flow-time relationships during breath cycle for model lungs with high compliance (ca. 200 mL/cm H_2_O) for various tidal volume divisions (lower outlet—branch with higher compliance; upper outlet—branch with lower compliance). Time 0–2 s represents inspiration phase, time 2–6 s represents expiration phase.

In the next step of this work, experimental studies were conducted in order to evaluate the effect of the system static compliance on the flow-time relationships during inspiration (time 0–2 s) and expiration phases (time 2–6 s). In order to simulate independent lung ventilation conditions, various tidal volume divisions between right and left lung were applied. The obtained results are summarized in Figure 5 (normal system compliance) and in Figure 6 (high system compliance).

According to Figure 5, significant differences between the flow distribution to the UO (branch with lower compliance, upper outlet) and LO (branch with higher compliance, lower outlet) appeared during both inspiration and expiration phases. This phenomenon was particularly remarkable for V_T_ division 1:3 (125 mL/375 mL) and 3:1 (375 mL/125 mL). Interestingly, in the lung which received the biggest amount of air two opposite flow-time dependences in the inspiration phase were observed—proportional for the lower outlet (higher compliance) and inversely proportional for the upper outlet (lower compliance). What should be underlined, in normal conditions the lines for flow-time relationship for lower and upper outlet should practically overlap with one another, no matter the tidal volume division 

Similar results were obtained for the model respiratory system with high system compliance (ca. 200 mL/cm H_2_O) as can be seen in Figure 6.

As one can easily notice in Figure 6, in all discussed V_T_ divisions, it was observed that flow-time relationship for lower outlet with higher compliance (blue line) was much closer to inlet flow-time relationship (black line) than in case of normal system compliance (Figure 5). The phenomenon was also particularly remarkable for V_T_ division 1:3 (125 mL/375 mL) and 3:1 (375 mL/125 mL). The possible explanation may be the compliance nonlinearity of model lung lobes. The Siemens test lung and anesthetic breathing bag had different elastic properties and their elasticity changed nonlinearly during the inspiration and expiration phases. The effect of two opposite flow-time dependences in the inspiration phase (Figure 5 and Figure 6), directly proportional for the lower outlet (higher compliance) and inversely proportional for upper outlet (lower compliance) can be explained in the following way—the branch with higher compliance had higher time constant (τ = Resistance × Compliance) than branch with lower compliance [26]. As a consequence, at the end inspiration phase, branch UO started expiring (in-phase) while branch LO was still inspiring (out-of-phase), a situation which caused the expired air from branch UO to fill in branch LO, simulating the pendelluft effect [25]. Higher system compliance enhanced the observed for normal compliance effect of gas transport between adjacent respiratory units with different compliances.

What is worth mentioning, described pendelluft flow may cause serious lung injury, particularly in patients undergoing high frequency oscillatory ventilation (HFOV) therapy. For ILV, due to the lower breath frequency than in case of HFOV, the phenomenon is probably less problematic, but certainly cannot be totally omitted from consideration. Even in ILV, the heterogeneous regional ventilation could increase the trapped gas and lead to volutrauma due to lung unit overdistention. Besides, as the pendelluft flow moves over the carina ridge, it causes a velocity gradient that induces high wall shear stress which may result in inflammation and rupture the lung epithelium layer [12,13].

### 3.3. Flow Distribution Analysis

To have a deeper insight to the lung ventilation abnormality, particularly the airways obstruction and alveolar overdistention [27,28], the flow–volume (Q–V) loops were analyzed in the next step. As one can easily notice in Figure 5 and Figure 6, the right and left lung reacts similarly during tests according to the similar trend of changes for studied flow-time relationships. Hence, for further analysis, only the left lung was considered not to double the discussed material.

**Figure 7:**
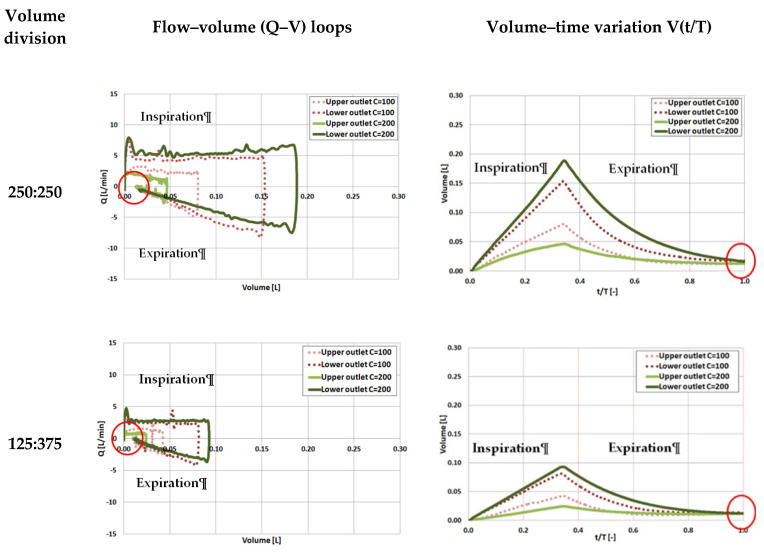

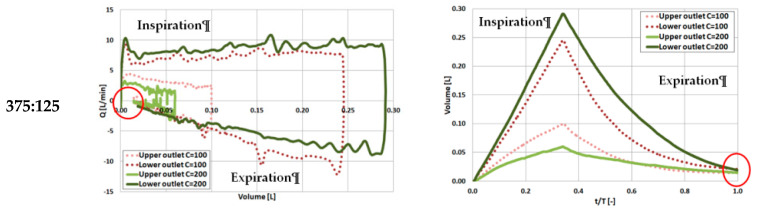
Flow–volume loops (Q–V) and volume–time variation (V(t/T)) during ventilation for model left lung with different system compliances (C = 100 and 200 mL/cm H_2_O) for various tidal volume divisions (lower outlet—branch with higher compliance; upper outlet—branch with lower compliance). *t/T* is normalized time, where *t* is the breath cycle time, *T*—is the total cycle time.

In Figure 7, there are depicted flow–volume (Q–V) loops and volume–time variations for three analyzed tidal volume division (250:250, 125:375, 375:125) and both system compliances (C = 100 and 200 mL/cm H_2_O).

In all cases presented in Figure 7, incomplete Q–V loops (marked with red circles) indicate that the gas volume did not completely return to the baseline at the end of expiration phase, signifying the possibility of trapped gas. This can be clearly seen also in volume–time variation graphs V(t/T)—all the lines do not reach the volume value equal zero (marked with red circles), as should occur in normal conditions with balanced compliances [25]. The residual gas volume could be attributed to the interregional gas exchange (pendelluft flow) during the cycle.

The higher system compliance was accompanied by increased discrepancies in the volumes that reached both lobes (comparison of the peak volume values obtained between lower and upper outlet for the same value of compliance, dashed or solid lines compared). The effect was enhanced with the increased tidal volume division. The possibility of gas entrapment and its amplification by the lobe compliance heterogeneity could lead to potentially serious overstretching of the alveolar region [29] and should be carefully observed in clinical conditions.

**Figure 8:**
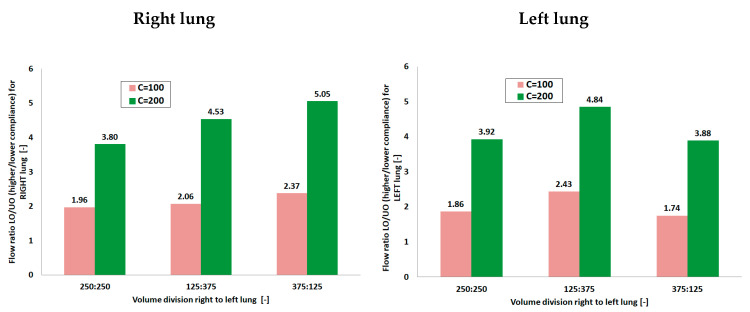
Flow distribution between branches with different compliances during ventilation for model lungs with different system compliances (C = 100 and 200 mL/cm H_2_O) for various tidal volume divisions (LO—branch with higher compliance; UO—branch with lower compliance).

To closely examine the role of lobe compliance on the breathing mechanics and interpret the observed behavior in the Q–V loops, global flow portioning between left and right lung as well as between local units (branches) were estimated as flow distribution ratio (FR). FR is defined as the ratio of the flow rates between the right and left lung lobes and is plotted in Figure 8. To calculate FR averaged maximum flow values obtained during the ventilation cycle were used.

Analyzing Figure 8, it can be seen that the flow characteristics and lobe ventilation were drastically affected by the differences in both lobes as well as by system compliance (comparison of FR values). For C = 100 mL/cm H_2_O a more balanced flow distribution of the gas between the left and right lobe was observed with a flow ratio equal to ca. 2 for almost all volume divisions. Increasing the system compliance to C = 200 mL/cm H_2_O was accompanied by a significant increase in the ventilation heterogeneity. A strong growing tendency of flow ratio in the case of right lung was also noticed with values from range 3.80–5.05. The dependency for the left lung turned out to be different than for the right lung—it may be supposed that differences in trachea anatomy and ETT asymmetry and position played the most important role in this phenomenon. Interestingly, Alzahrany and Banerjee [30] observed the influence of gas density on the flow distribution with increasing density resulting in increasing ventilation heterogeneity. They attributed this fact to the readjustment of the flow velocity distribution and turbulence due to the change in the gas density and viscosity. Consequently, a more balanced flow distribution was achieved by reducing the gas density. Considering this, in case of results presented in the current work, it may be stated that higher system compliance caused more unstable flow, thus resulting in increasing ventilation heterogeneity.

Obtained results prove the clinical importance of experimental studies in models close to reality, imitating the anatomy and pathologies of the respiratory system. In this context, further work is also needed regarding the influence of double-lumen ETT on the therapeutic aerosol deposition in model diseased lungs with different compliances during ILV procedure. It is a crucial step to the enhancement of the effectiveness of drug delivery by inhalation, which is currently rather unsatisfactory particularly in diseased lungs [31,32].

## 4. Conclusions

Summarizing, the main aim of this study was to evaluate the influence of double-lumen ETT on the gas transport and mixing in the anatomically accurate 3D-printed model of the bronchial tree, with lung lobes of different compliances, using various ventilation modes imitating independent lung ventilation. The computer model of the upper part of the bronchial tree, based on CT scans, was obtained from Respiratory Drug Delivery, processed and printed by the dual-extruder FFF 3D printer. The realistic geometry of airways is essential for evaluating the pulmonary function and respiratory disease, not only in case of CFD studies [33] but also experimental tests as was proved in this work. Thanks to an accurate model of the upper part of the bronchial tree, it was possible to record all the described above effects and flow phenomena. Firstly, the measured resistance increased six times in the presence of double-lumen ETT as compared to the model system without the tube. Secondly, discrepancies in the flow distribution to the less and more compliant lobe were more significant for the airways with double-lumen ETT. The effect was dependent on the tidal volume division (particularly remarkable for V_T_ division 125:375 and 375:125) between lungs and greatly enhanced by the increase in system compliance. Observed possibility of gas entrapment and its amplification by the lobe compliance heterogeneity could lead to potentially serious overstretching of the alveolar region [29] and should be carefully observed in clinical conditions during ILV procedure.

## Figures and Tables

**Table 1:** Printing settings.

Parameter	Values
Model Material	PLA (3DGence)
Support Material	BVOH (Verbatim)
Printing temperature	200 (°C)
Infill percent	20 (%)
Infill pattern	Zig Zag
Side walls	2
Top walls	6
Bottom walls	4
Bed adhesion	Raft
Layer height	0.15 (mm)
Printing time	9 h 50 min

**Table 2:** Experimental variants.

Parameter	Values	Unit
System static compliance, C	ca. 100 (normal) ca. 200 (high, pathological, e.g., emphysema)	mL/cm H_2_O
Tidal volume, V_T_	500	mL
Inspiration-expiration ratio, I:E	1:2.0	-
Duration of the breath cycle, T	6	S
Breath frequency, f	10	1/min
Tidal volume (V_T_) division (right to left lung)	1:1 (250 mL:250 mL) 1:3 (125 mL:375 mL) 3:1 (375 mL:125 mL)	-

**Table 3:** Pressure and flow ratios for measurements with and without double-lumen ETT; LO—lower outlet (high compliance lobe), UO—upper outlet (low compliance lobe).

V_T_ Division	Resistance (cm H_2_O/L/s)	Pressure Ratio LO/UO Right Lung	Pressure Ratio LO/UO Left Lung	Flow Ratio LO/UO Right Lung	Flow Ratio LO/UO Left Lung
1:1 with ETT	**15**	1.01	1.02	**1.96**	**1.86**
1:1 no ETT	**2.5**	1.02	1.00	**1.85**	**1.68**

## Data Availability

The data can be obtained on request by e-mail contact with corresponding author.
